# An Effective Method for Acute Vagus Nerve Stimulation in Experimental Inflammation

**DOI:** 10.3389/fnins.2019.00877

**Published:** 2019-08-27

**Authors:** April S. Caravaca, Alessandro L. Gallina, Laura Tarnawski, Kevin J. Tracey, Valentin A. Pavlov, Yaakov A. Levine, Peder S. Olofsson

**Affiliations:** ^1^Laboratory of Immunobiology, Center for Bioelectronic Medicine, Department of Medicine, Solna, Karolinska Institutet, Stockholm, Sweden; ^2^Center for Biomedical Science and Bioelectronic Medicine, The Feinstein Institute for Medical Research, Manhasset, NY, United States; ^3^Setpoint Medical, Inc., Valencia, CA, United States

**Keywords:** Bioelectronic Medicine, vagus nerve stimulation, neural reflex, inflammation, inflammatory reflex, peripheral nerve, neural control

## Abstract

Neural reflexes regulate inflammation and electrical activation of the vagus nerve reduces inflammation in models of inflammatory disease. These discoveries have generated an increasing interest in targeted neurostimulation as treatment for chronic inflammatory diseases. Data from the first clinical trials that use vagus nerve stimulation (VNS) in treatment of rheumatoid arthritis and Crohn’s disease suggest that there is a therapeutic potential of electrical VNS in diseases characterized by excessive inflammation. Accordingly, there is an interest to further explore the molecular mechanisms and therapeutic potential of electrical VNS in a range of experimental settings and available genetic mouse models of disease. Here, we describe a method for electrical VNS in experimental inflammation in mice.

## Introduction

Excessive inflammation plays an important role in the pathogenesis of a range of common and debilitating acute and chronic diseases, including septic shock, rheumatoid arthritis, inflammatory bowel disease, and cardiovascular disease (Nathan and Ding, 2010). Therapeutic interventions to reduce cytokine levels and attenuate inflammation significantly improves symptoms and outcomes in many of these diseases (Feldmann, 2002; Ridker et al., 2017). Anti-cytokine drugs have shown success in clinical trials, but are costly and not always effective and/or suitable for patients that suffer from diseases characterized by excessive inflammation (Hansel et al., 2010; Li et al., 2017; Lin et al., 2019).

Discoveries on the neural reflex control of inflammation, particularly the neurophysiological and molecular mechanisms of vagus nerve regulation of systemic cytokine levels in the “*inflammatory reflex*” (Tracey, 2015), have made it possible to consider electrical vagus nerve stimulation (VNS) in the treatment of inflammation and inflammatory diseases (Pavlov and Tracey, 2015; Eberhardson et al., 2019). This neuro-immune reflex mechanism has an afferent arc that senses inflammation and injury, and an efferent arc that regulates cytokine production and release (Olofsson et al., 2012). The first clinical trials implementing these discoveries using implanted nerve stimulators to activate the inflammatory reflex and treat chronic inflammation have reported encouraging results on amelioration of disease activity score (Koopman et al., 2016). Based on these observations, further exploration of the effects of electrical VNS on the immune system in the many available mouse models of inflammatory diseases is of great interest.

The vagus nerve is a cranial nerve and contains sensory and motor fibers wrapped in a sheath of connective tissue. Establishing an electrical interface with the vagus nerve that produces consistent stimulation in mouse models of inflammatory diseases requires standardization in dissection, electrode placement, and charge delivery. The methods for VNS in mouse models of inflammation used in published studies vary significantly, which may complicate interpretation of reported findings (Inoue et al., 2016; Meneses et al., 2016; Le Maître, Revathikumar et al., 2017). Sources of variation include differences in the dissection method, the physical interface with the nerve, charge delivery, sham treatment, anesthesia and stimulation monitoring (Borovikova et al., 2000; Huston et al., 2006; Rosas-Ballina et al., 2008).

Wide-spread availability of a simple, consistent, reproducible method for VNS would likely facilitate progress in the field (Kwan et al., 2016). Here, we describe a method for performing VNS for study of experimental inflammation that in the experience of the authors yields consistent and reproducible results across laboratories (Olofsson et al., 2015; Caravaca et al., 2017; Tarnawski et al., 2018).

## Methods and Results

### Ethics Statement

This study and all experimental protocols were approved by the Stockholm Regional Board for Animal Ethics (Stockholm, Sweden).

### Animals

We used male BALB/c and C57Bl/6J mice purchased from Charles River Laboratories (median age was 10 weeks and the range 10–40 weeks). The animals were housed in a laboratory environment on a 12 h light/dark cycle, with *ad libitum* access to food and water.

### Statistical Analysis

Differences between experimental groups were analyzed using unpaired two-tailed Student’s *t*-test or one-way ANOVA as appropriate. Data are presented as mean ± SEM. *p* ≤ 0.05 was considered significant. Prism 8 (GraphPad software, San Diego, CA, United States) was used for analyses.

### Titration of Inflammatory Insult

**FIGURE 1:**
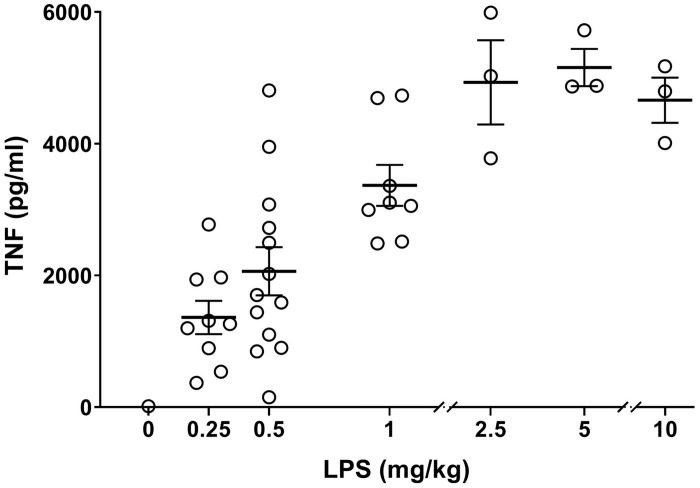
Establishing TNF dose response in endotoxemia. Alert mice were injected intraperitoneally with 0–10 mg/kg of endotoxin and blood was collected by cardiac puncture 90 min later. Serum TNF levels, measured by ELISA, are plotted as mean ± SEM. *n* = 3–13 mice per group.

For each batch of lipopolysaccharide (LPS), the dose was titrated for endotoxemia experiments. Bacterial LPS from *Escherichia coli*, O111:B4 (Manufacturer #L2640, Lot # 097M4041V) (Sigma-Aldrich, MO, United States) was prepared to a concentration of 5 mg/ml (0.5% of LPS in MilliQ water) and aliquots were stored at –20°C. Prior to use, aliquots were thawed and then sonicated (Branson B200, Connecticut, United States) for 30 min to dissolve and disaggregate LPS in the solvent (Ogawa and Kanoh, 1984). Mice were injected intraperitoneally with 0, 0.25, 0.5, 1, 2.5, 5, and 10 mg/kg of LPS and then euthanized using carbon dioxide asphyxiation 90 min after LPS injection (Rosas-Ballina et al., 2008). This time point was chosen because in rodent endotoxemia, serum TNF reaches its maximum concentration of TNF between 60 and 90 min after LPS injection (Huston et al., 2006). Blood was immediately collected via cardiac puncture. Samples were incubated at 1 h at room temperature and then centrifuged at 2700 × *g* for 7 min (Eppendorf, Hamburg, Germany). To remove cells from the serum, the supernatant was transferred to a new tube and centrifuged a second time at 10,600 × *g* for 1 min, and serum was retained. TNF levels in serum were analyzed by an enzyme-linked immunoabsorbant assay (ELISA) kit (R&D Systems, MN, United States). In this experiment, TNF levels reached a plateau at endotoxin doses ≥ 2.5 mg/kg. Accordingly, using this specific batch of endotoxin, approx. 0.25 – 1 mg/kg LPS was considered suitable for studying effects of interventions on serum TNF levels in the physiological range (Figure 1). Of note, in our previous studies with a different batch of endotoxin (Tarnawski et al., 2018), up to 8 mg/kg of LPS was used (Borovikova et al., 2000; Huston et al., 2007) and suppression of serum TNF levels was observed.

### Equipment Setup

**FIGURE 2:**
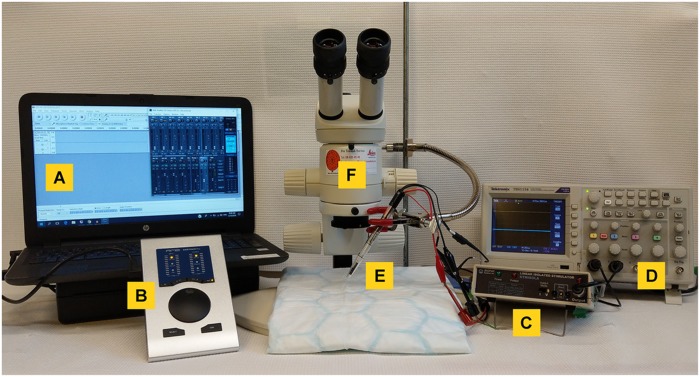
Equipment setup for mouse vagus nerve stimulation. **(A)** A computer with a waveform editing program, connected to **(B)** a digital-to-analog interface to deliver the pulse output and a **(C)** voltage-to-constant current converter. To visualize voltage output an **(D)** oscilloscope was used. **(E)** A custom-built bipolar electrode was used to connect to the vagus nerve. Surgery was performed under a **(F)** stereotactic microscope.

The setup for mouse VNS is shown in Figure 2 and includes a computer (CAN ICES-3(B)/NMB-3(B), HP, California, United States) (Figure 2A) and a digital-to-analog interface for pulse generation (RME Fireface UFX or RME Babyface Pro, Audio AG, Haimhausen, Germany) (Figure 2B), a voltage-to-current converter (STIMSOLA, Biopac, CA, United States) (Figure 2C), an oscilloscope (Tektronix, Oregon, United States) (Figure 2D) to observe and record electrical signals, and a custom-built electrode for interfacing with the nerve (Figure 2E). A stereo microscope, preferably on a balanced swivel and arm, is recommended for surgery.

Different equipment and stimulator setups were evaluated, including a number of commercially available devices for nerve stimulation. In our experience, systems capable of delivering a suitable pulse at sufficiently high constant current work well to activate the inflammatory reflex (data not shown). Here, we used open source software (Audacity)^1^ and a high-quality audio interface to generate the desired voltage. The voltage was fed through a voltage-to-constant current interface. The electrical output of the setup at a range of resistive loads was recorded (Supplementary Figure 1) to verify the performance of the setup.

### Electrode Construction

**FIGURE 3:**
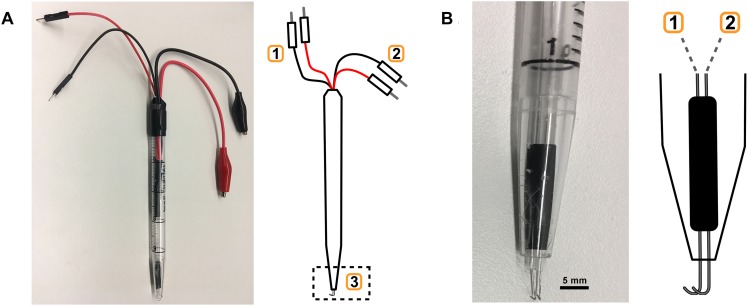
Electrode fabrication. **(A)** Custom-built bipolar electrode with monitoring (1) and stimulation (2) leads, and the hook electrode (3). **(B)** Close-up of hook electrode (3) with connections to (1) and (2).

The hook-type electrode described here was made from 0.25 mm platinum-iridium (Pt:Ir; 90:10%) (Figure 3). We connected a bipolar hook electrode with two pairs of connecting wires to the stimulator and oscilloscope, respectively (Figure 3). The spacing between the two electrode tips was fabricated to approximately 0.5 mm (Olofsson et al., 2015).

Platinum–iridium and silver electrodes enable low-impedance electrical interfaces to the vagus nerve with limited toxicity to cells and tissues (Geddes and Roeder, 2003; Navarro et al., 2007). Silver electrodes can be used in the experimental setup described here as a cost efficient alternative to platinum–iridium (Supplementary Figure 2).

### Surgical Tools

**FIGURE 4:**
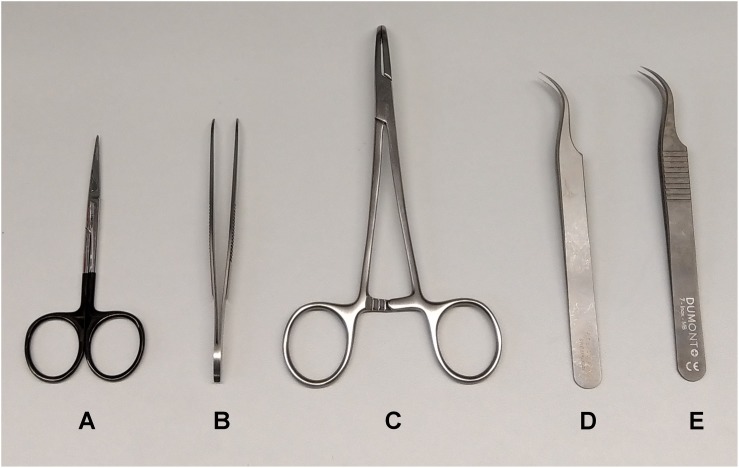
Surgical tools for vagus nerve isolation. **(A)** Serrated micro dissection scissors. **(B)** Curved dressing forceps. **(C)** Curved hemostatic forceps. **(D)** Non-serrated standard curved forceps. **(E)** Non-serrated fine curved forceps.

Isolation of the vagus nerve requires delicate surgical manipulations, and appropriate instruments are key. Here, fine serrated micro dissection scissors (Agntho’s #14058-09) (Figure 4A) and curved dressing forceps with serrated tips (Agntho’s #11051-10) (Figure 4B) for handling skin were used. A pair of curved hemostatic forceps (Agntho’s #13013-14) (Figure 4C) were used to retract skin and expose the surgical site. We used a pair of non-serrated standard curved forceps (Agntho’s, #0303-7-PS) (Figure 4D) to dissect apart salivary glands, and surrounding tissues. We then used a pair of fine curved surgical dissection forceps (Agntho’s, #0208-7-PS) (Figure 4E) for manipulations involving the carotid artery and vagus nerve. Instruments were cleaned and disinfected before and after use between experiments. Extra care is required for fine tools as the tips are very fragile. Damaged tips can affect the quality of the isolation and cause injury to the nerve.

### Surgical Procedure: Isolation of the Vagus Nerve

**FIGURE 5:**
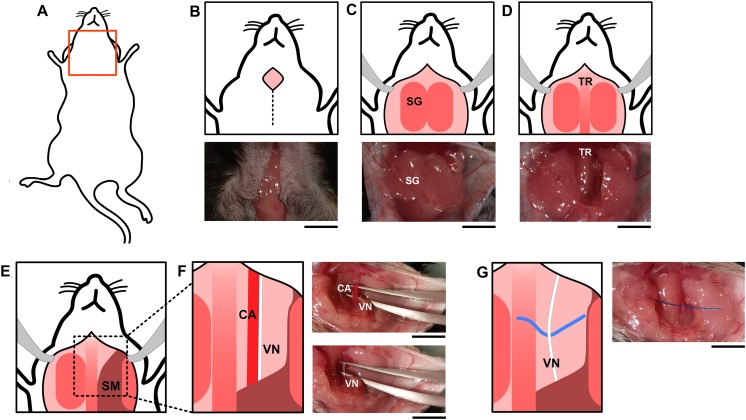
Surgical vagus nerve isolation for electrical stimulation. **(A)** The neck area of the mouse was shaved and swabbed with ethanol. **(B)** A midline cervical incision was made, exposing the **(C)** salivary glands (SG) and **(D)** trachea (TR). Subcutaneous tissues between the **(E)** sternomastoid (SM) and sternohyoid muscles along the trachea were separated using blunt dissection which reveals the **(F)** common carotid artery (CA) and the cervical vagus nerve (VN). The vagus nerve and the carotid artery **(F)** are located parallel to each other and were separated using curved forceps with tips facing upward. One set held both the vagus nerve and carotid artery, and the other was used to separate the two structures. The forceps were then carefully opened and closed repeatedly to progressively detach the vagus nerve from the carotid artery. **(G)** A piece of polypropylene suture was placed under the nerve to facilitate electrode placement. The black scale bar indicates 5 mm.

An absorbent mat with plastic backing was placed over the surgical area. Anesthesia was induced using isoflurane at 3% in a 1:1 mixture of oxygen and air. Following induction of anesthesia, mice were placed on the surgical mat in the supine position (Figure 5A), and maintained at 1.5% isoflurane in a 1:1 oxygen:air mixture. The neck area was shaved and the loose fur removed with a gauze or adhesive tape. Masking tape was used to secure the paws in a fixed position to the surgical mat. The shaved neck area was swabbed with 70% ethanol. A ventral midline cervical incision was made between the mandible and sternum (Figure 5B). Subcutaneous tissues were separated using blunt dissection and retracted laterally with hemostatic forceps to expose the salivary glands (Figure 5C). The two lobes of the salivary glands were separated by simultaneously pulling them apart, away from the midline, to reveal the sternomastoid and sternohyoid muscles along the trachea (Figure 5D). Blunt dissection to either the right or left revealed the common carotid artery and the cervical vagus nerve forming a neurovascular bundle, or carotid sheath (Figure 5E). The vagus nerve was isolated away from the carotid artery and the surrounding connective sheath tissue (Figure 5F). A 1–2 cm piece of polypropylene suture was placed under the nerve to facilitate electrode placement (Figure 5G).

**FIGURE 6:**
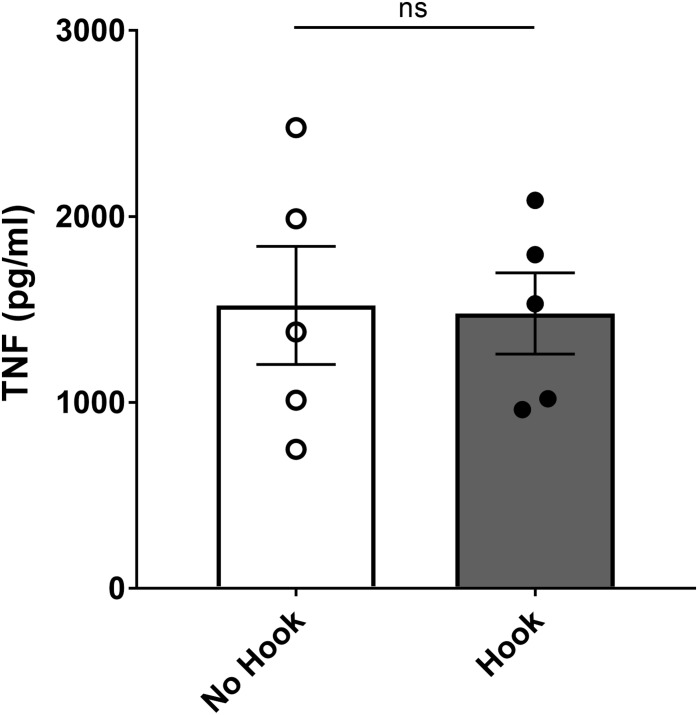
No significant effect on endotoxemic serum TNF level by application of hook electrode. Mice were subjected to sham surgery with or without physical suspension of the vagus nerve on the hook electrode. After full recovery from anesthesia, mice were injected with endotoxin and blood collected 90 min later. Serum TNF was analyzed by ELISA. Results are shown as mean ± SEM, *n* = 5 mice per group from three distinct experiments; ns, not significant (unpaired, two-tailed Student’s *t*-test).

For sham surgery, the ventral midline cervical incision was performed, subcutaneous tissues were separated using blunt dissection, and salivary glands were separated (Figures 5A–C). It has been reported that mechanical stimulation of the vagus nerve reduces serum TNF levels compared to sham in mouse endotoxemia (Huston et al., 2004). To study whether the described isolation method and manipulation of the vagus nerve reduces TNF in mouse endotoxemia *per se*, we compared serum TNF levels between animals where the isolated vagus nerve was suspended on the electrode without electrical stimulation (Figures 5A–G + nerve suspension on hook) or left untouched on the carotid artery (Figures 5A–D). We observed no significant difference in serum TNF between these two groups (Figure 6). We observed no significant difference in serum TNF level in the sham surgery mice with or without vagus nerve isolation from the sheath (Figure 6). This observation suggests that manipulation of the vagus nerve in a careful manner does not necessarily elicit activation of the cholinergic anti-inflammatory pathway.

After stimulation, the electrode was removed, and in both the stimulation and sham surgery group, tissues were restored to their original position, and the incision sutured (Silk, 4-0, FS-2 needle) or stapled with wound clips (stainless steel, 9 mm). During prolonged anesthesia, it is advisable to maintain core temperature at a physiological level using a rectal temperature probe and heating pad under the absorbent surgical mat during surgery, to prevent hypothermia. It is also recommended to place a heating pad under a clean cage for the mouse during recovery, however, precautions should be taken to ensure the animals are not overheated during recovery by covering a portion of the bottom of the cage so animals can move away from heated areas as they desire.

### Vagus Nerve Stimulation

**FIGURE 7:**
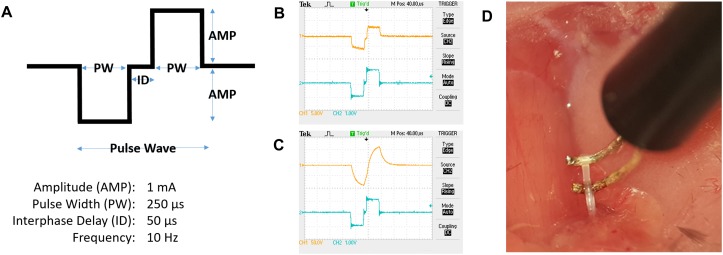
Electrical stimulation of the cervical vagus nerve. **(A)** Schematic representation of a pulse wave. **(B)** Oscilloscope tracing of voltage output from the digital to analog interface (blue tracing, scale 1 V/square) at the desired impedance and voltage measured over the electrode leads (orange tracing, scale 5 V/square). **(C)** Example of tracing with electrode-nerve interface with a high impedance level (orange tracing, scale 50 V/square). **(D)** The isolated vagus nerve segment resting on custom-built hook electrodes.

Constant current stimulation was applied to the nerve at 1 mA, 250 μs biphasic pulse, 50 μs interphase delay, at 10 Hz for 5 min (Figure 7A) (Olofsson et al., 2015). A charge-balanced biphasic square waveform for stimulation was used (Lilly et al., 1955). A charge-balanced pulse generates less tissue and electrode surface damage compared to unbalanced charge delivery (Harnack et al., 2004). Mice in the sham group were subjected to surgery, but not to electrical stimulation. Constant current was maintained by the constant current stimulator setup, as visualized using the voltage output on an oscilloscope (Figures 7B,C). In our stimulations, we visualized the output from the stimulator (Figure 7B; blue tracing represents voltage output from digital to analog interface), and the voltage across the electrodes at the tissue interface (Figure 7B; orange tracing represents electrode-nerve interface).

Voltage drop across the resistance was calculated from the oscilloscope tracing and is based on Ohm’s Law (*Z* = *V*/*I*). *Z* is the impedance in ohms (Ω), *V* is the observed voltage across the electrodes, and *I* is the programed current delivered by the stimulator.

First phase cathodic stimulation was used because nerve activation occurs at lower current than during anodic stimulation (McIntyre and Grill, 1999; Basser and Roth, 2000). We have previously reported constant current levels sufficient to activate the inflammatory reflex and reduce TNF in acute experimental endotoxemia (Olofsson et al., 2015).

Consistent current delivery requires that the electrical path is confined to the nerve and isolated from the surrounding tissues. Even with insulated wiring the curvature of the hook wires may be exposed to surrounding tissue and extracellular liquid, offering an alternative electrical path. Caution must be exercised when suspending the nerve on the hook electrodes (Figure 7D) to avoid mechanical stretch injury that may cause aberrant or adverse effect.

### Visual Observations

Non-specific muscle activation is a sign of unwanted current delivery to tissues outside the nerve. The vagus nerve and the electrode need to be sufficiently separated from surrounding tissues and fluids to avoid current leakage. Of note, twitching of specific laryngeal muscles is observed during stimulation, as the vagus nerve supplies motor nerve fibers to this area.

An oscilloscope was used during stimulation to continuously measure the electrical output as it fluctuates with variations in impedance. Changes in the oscilloscope tracings during stimulation should be observed and noted, as large deviations in the expected voltage tracing can reflect current leaks, faulty electrodes, problems with nerve-electrode contact, and other reasons for suboptimal charge delivery.

### Vagotomy

Disruption of vagus nerve signaling aggravates systemic inflammation in experimental models of disease (Borovikova et al., 2000; van Westerloo et al., 2006; van Maanen et al., 2009). To study disruption of vagus nerve signaling, unilateral cervical vagotomy can be used. The surgical procedure is similar to the approach for VNS, as previously described herein. After isolation of the vagus nerve, a piece of 1–2 cm polypropylene suture was placed under the nerve. Forceps were used to carefully and gently lift and hold the nerve suspended. Cuts were made above and below the forceps grip in order to remove a segment of nerve approximately 2–3 mm in length. 2–3 mm is a minor discrepancy in terms of measurement used in vagotomy, and in our experience it is sufficient for acute experiments as the nerve remains severed (data not shown). After resection, salivary glands and tissues were restored to position and the skin was sutured (Silk, 4-0, FS-2 needle) or stapled with wound clips (stainless steel, 9 mm).

### Endotoxemia

The endotoxin model of systemic inflammation is commonly used, and experimental murine endotoxemia is well established (Fink and Heard, 1990; Remick et al., 2000; Lewis et al., 2016). LPS binds to toll-like receptor 4 (TLR4) which is present on a number of cells such as monocytes and macrophages and promotes the secretion of pro-inflammatory cytokines such as TNF. Mice were allowed to recover for a minimum of 1 h from anesthesia and surgery before intraperitoneal administration of endotoxin, and longer recovery periods can be used (Tarnawski et al., 2018). The half-life of ketamine and xylazine in rodents can be as long as 2 h (Veilleux-Lemieux et al., 2013). Anesthesia has been reported to delay the inflammatory response (Fuentes et al., 2006). It is important for experimental consistency in the inflammatory response that mice recover from anesthesia before the inflammatory agent is administered.

### Sample Processing and Cytokine Measurement

**FIGURE 8:**
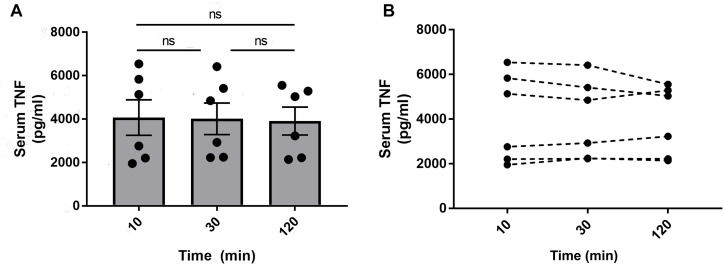
Serum TNF in blood stored at room temperature for 10, 30, or 120 min. Alert mice were injected intraperitoneally with 0.25 mg/kg endotoxin. Mice were euthanized after 90 min and blood was collected via cardiac puncture. The blood was aliquoted in three separate tubes and stored at room temperature for 10, 30, or 120 min before centrifugation to isolate serum. TNF was then measured by ELISA and **(A)** plotted as mean ± SEM and **(B)** as individual TNF values for each mouse, *n* = 6 per group from one experiment; ns, not significant, one-way ANOVA followed by Tukey’s test.

**FIGURE 9:**
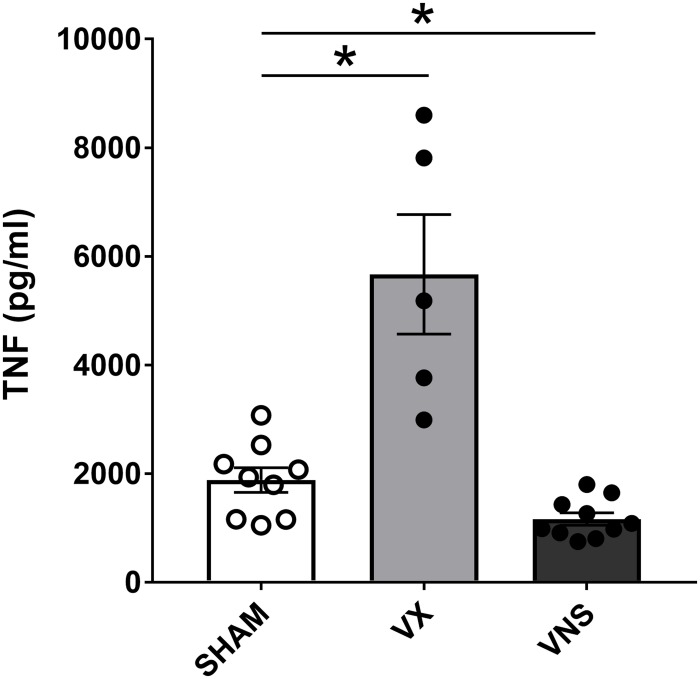
Stimulation or ablation of cervical vagus nerve signaling alters serum TNF in endotoxemia. Vagus nerve stimulation (VNS) or vagotomy (VX) was performed under anesthesia. Mice were allowed to recover fully and then injected with LPS intraperitoneally. Blood was collected 90 min post-injection and serum TNF was analyzed by ELISA. *n* = 5–10 mice per group from five distinct experiments. ^∗^*p* = 0.05, one-way ANOVA followed by Dunnett’s *post-hoc* test.

There are multiple steps involved in blood collection and sample processing, so we investigated whether delayed separation of whole blood and serum concentrations of TNF varied for up to 120 min. Mice were euthanized 90 min after injection of endotoxin and blood was collected via cardiac puncture using a 1 mL syringe fitted with a 23 gauge needle. The needle was then removed from the syringe to prevent hemolysis and the blood from each mouse was dispensed into three aliquots. Subsequently, the aliquots were left for 10, 30, or 120 min at room temperature before centrifugation. After the two-step centrifugation process as previously mentioned, the serum was transferred to Eppendorf tubes, frozen on dry ice, and transferred to –80°C for long term storage. Cytokine levels were measured in serum by ELISA or multiplex assay kit (Meso Scale Discovery, MD, United States) according to manufacturer protocols. There was no significant difference in TNF levels between the groups from the different incubation times (Figure 8). The serum TNF level in samples from individual mice was stable across the incubation period at room temperature (Figure 8). However, in our experiments, we do standardize the time between collection and serum/plasma collection for each batch of samples. In line with previous observations (Borovikova et al., 2000; Olofsson et al., 2015), serum TNF levels were significantly reduced in endotoxemic mice subjected to electrical VNS, while unilaterally vagotomized endotoxemic mice showed significantly elevated serum TNF levels compared to sham (Figure 9).

## Discussion

Here, we define a method for VNS in experimental mouse endotoxemia. The significance of endotoxin titration, electrode fabrication, surgical vagus nerve isolation, and electrical stimulation and monitoring is described, as well as key points in experimental procedures.

There is an increasing interest in neural reflex control of inflammation, and electrical VNS in the context of experimental inflammation. However, the details in implementation of VNS for activating the inflammatory reflex vary considerably in the literature. In our experience, certain key features in the procedures are important to avoid inconsistent results across experiments and laboratories. Specifically, the induction of inflammatory insult should be kept within a physiological range, dissection of the vagus nerve must be uniform, and charge delivery to the nerve controlled. In addition, the particulars of sample processing are important.

It is crucial to be aware that even minor trauma to the vagus nerve, which can easily occur during the surgical isolation and suspension of the nerve on the electrode, can significantly affect physiology. Of note, stretching and compression during handling can cause physical stress that interferes with nerve function. Trauma may cause firing of action potentials or impairment of electrical activation with obvious significant effects on the consistency of experimental results. In our experience, the quality and condition of surgical tools are paramount along with proper training of the animal surgeon.

It is necessary to titrate the endotoxin to determine a suitable dose within the dynamic range of TNF response. In the case of endotoxin, there is commonly a significant lot-to-lot variation in potential to induce inflammation. Each batch must therefore be evaluated to find suitable dosing that produces sufficient inflammation within reasonable physiological limits (Thompson and Chesher, 2018). In this study, we found that the serum TNF level 90 min after endotoxin administration plateaued above 2.5 mg entodoxin/kg body weight for the used lot of endotoxin. Accordingly, doses from 0.25 to 1 mg/kg bodyweight produced a significant TNF response within the physiological range. Different strains of animals and animal species may vary in tolerance and measurable TNF response to LPS, which is another reason to titrate the suitable LPS dose for each specific experimental setup (Thomas et al., 2014).

A key component of consistent VNS is the integrity of the electrode interface with the vagus nerve, and a reliably stable charge delivery by a high-quality voltage-to-constant current converter. For electrode fabrication, a range of materials are suitable, including platinum–iridium (Olofsson et al., 2015), silver (Inoue et al., 2016), and tungsten–titanium (Caravaca et al., 2017). The goal is to create a non-toxic, efficient and stable interface between the stimulation equipment and the nerve. Electrodes may oxidize with use, so it is important to monitor the integrity of the interface over time. Long-term exposure to phosphate buffered saline (PBS) of various metal contacts can also influence electrode integrity (Howlader et al., 2015). A convenient way to monitor interface integrity is to measure interface impedance during the electrical stimulation. This can be achieved by connecting an oscilloscope and measuring the voltage across the electrode continually through the stimulation. Here, we observed voltages over the electrode around 5 V which at 1 mA current corresponds to an impedance of 5 kΩ, which in our experience is suitable for electrical VNS to activate the inflammatory reflex.

Optimal electrical stimulation parameters for activation of the inflammatory reflex are not known, but some empirical data on suitable stimulation settings are available. We have previously investigated a range of constant current stimulus parameters in both mice and rats, which we used to select the stimulation settings in this study, i.e., 1 mA current, 250 us pulse width at 10 Hz for 5 min (Olofsson et al., 2015). Going forward, it will be important to further optimize stimulation parameters for activation of the inflammatory reflex in experimental inflammation.

In line with previous observations (Borovikova et al., 2000; Olofsson et al., 2015; Tarnawski et al., 2018), serum TNF levels were significantly reduced in endotoxemic mice subjected to electrical VNS, while unilaterally vagotomized endotoxemic mice showed significantly elevated serum TNF levels compared to sham. Interestingly, it has been observed by others that physical manipulation of the vagus nerve may activate the inflammatory reflex (Huston et al., 2006). In itself, the careful physical placement of the cervical vagus nerve on the electrodes in this study did not significantly change serum TNF levels in endotoxemia. This is achieved by employing a deliberately careful surgical technique to reduce the physical perturbation of the nerve, suggesting that mechanical manipulation of the vagus nerve does not necessarily elicit activation of the inflammatory reflex.

We observed that blood incubated between 10 and 120 min at room temperature did not significantly affect levels of measured TNF in this study, at least not in the range ≈2–6 × 10^3^pg/mL. While this observation may tempt investigators to streamline the sample handling process by reducing the need for careful timing of the serum isolation procedure, we still advocate to practice consistent timing of sample processing until more data is available.

In conclusion, we provide a method for electrical stimulation of the vagus nerve in experimental inflammation in mice.

## Data Availability

The datasets generated for this study are available on request to the corresponding author.

## Author Contributions

AC, YL, AG, VP, KT, and PO contributed to the conception and design of the study. AC and AG performed the experiments, analyzed the data, and wrote the manuscript with supervision from YL, PO, VP, and LT. LT designed the figures. All authors provided the critical feedback, and edited and finalized the manuscript.

## Conflict of Interest Statement

YL is employed by SetPoint Medical, Inc., Valencia, CA, United States. The remaining authors declare that the research was conducted in the absence of any commercial or financial relationships that could be construed as a potential conflict of interest.

## Footnotes

1 www.audacityteam.org
