# Supplementary figures and images for: An Effective Method for Acute Vagus Nerve Stimulation in Experimental Inflammation
Source: Front Neurosci. 2019 Aug 27;13:877. doi: 10.3389/fnins.2019.00877 (PMC6736627; doi:10.3389/fnins.2019.00877)

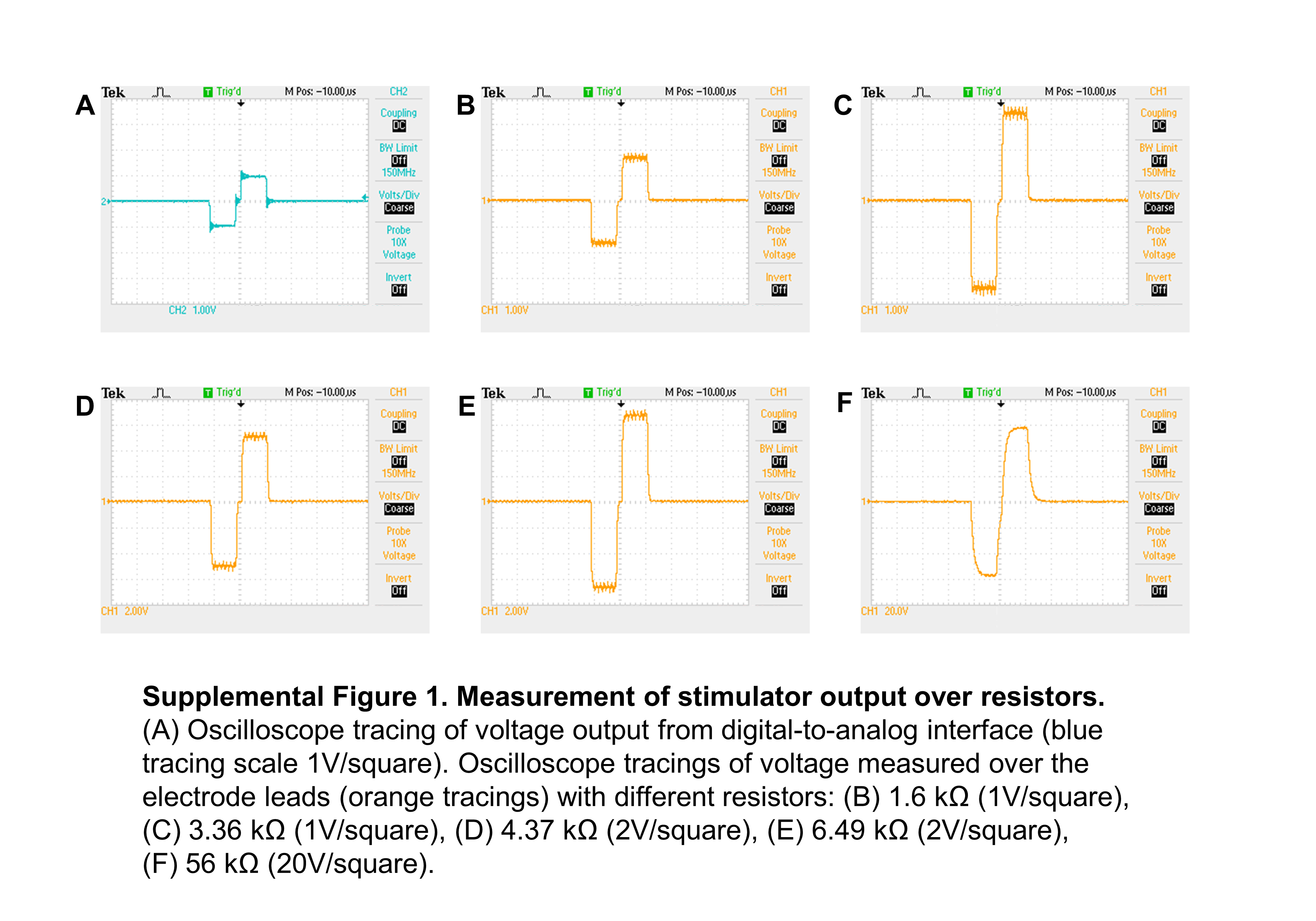

Supplement: Supplementary file 1 [file Image_1.tif]

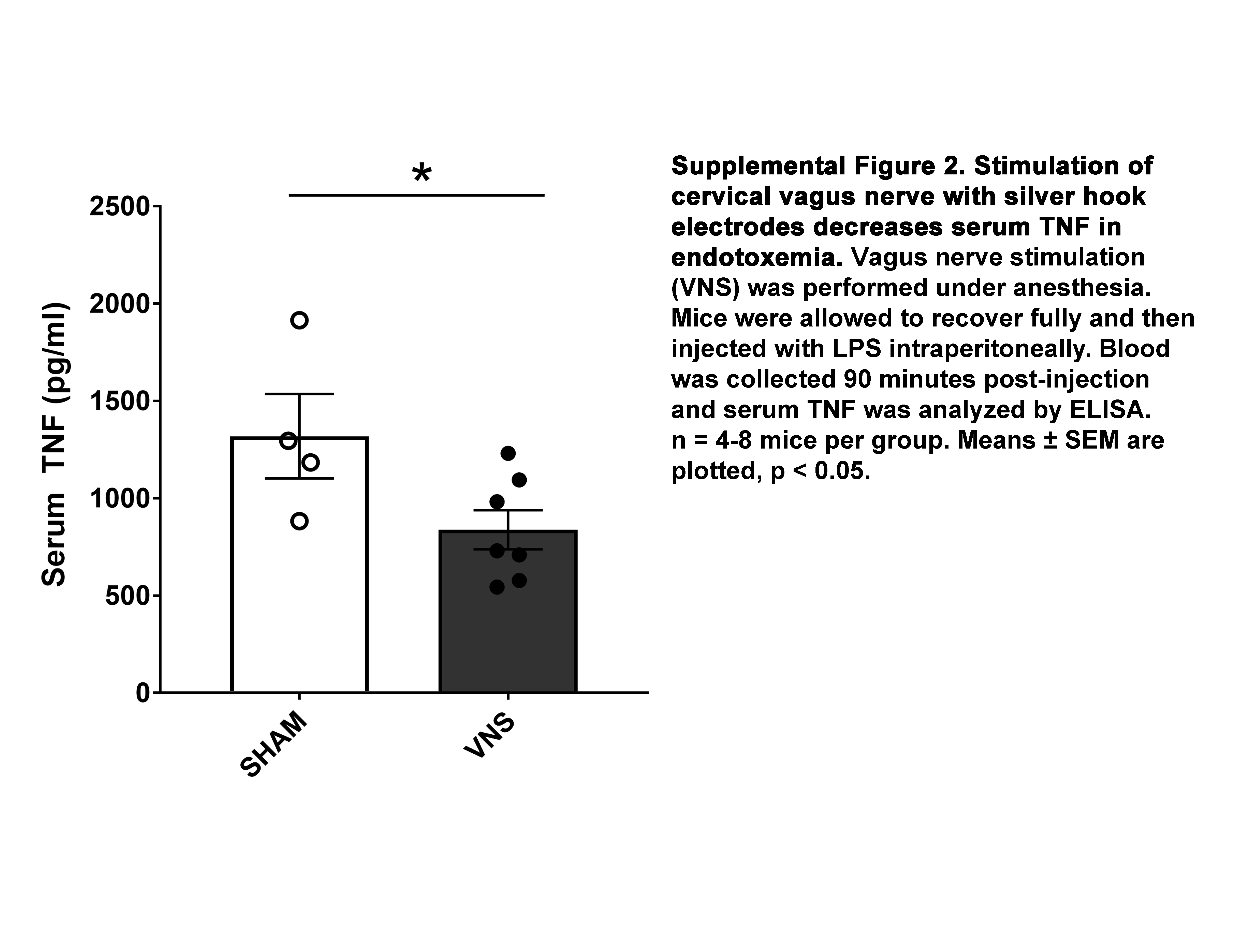

Supplement: Supplementary file 2 [file Image_2.tif]
